# Public Awareness of Colorectal Cancer Screening: Knowledge, Attitudes, and Interventions for Increasing Screening Uptake

**DOI:** 10.1155/2014/425787

**Published:** 2014-03-05

**Authors:** Antonio Z. Gimeno Garcia, Noemi Hernandez Alvarez Buylla, David Nicolas-Perez, Enrique Quintero

**Affiliations:** ^1^Servicio de Aparato Digestivo, Hospital Universitario de Canarias, Unidad de Endoscopia, La Laguna, 38320 Tenerife, Spain; ^2^Departamento de Gastroenterología, Hospital Universitario de Canarias, Unidad de Endoscopia, Ofra s/n, La Laguna, 38320 Tenerife, Spain

## Abstract

Colorectal cancer ranks as one of the most incidental and death malignancies worldwide. Colorectal cancer screening has proven its benefit in terms of incidence and mortality reduction in randomized controlled trials. In fact, it has been recommended by medical organizations either in average-risk or family-risk populations. Success of a screening campaign highly depends on how compliant the target population is. Several factors influence colorectal cancer screening uptake including sociodemographics, provider and healthcare system factors, and psychosocial factors. Awareness of the target population of colorectal cancer and screening is crucial in order to increase screening participation rates. Knowledge about this disease and its prevention has been used across studies as a measurement of public awareness. Some studies found a positive relationship between knowledge about colorectal cancer, risk perception, and attitudes (perceived benefits and barriers against screening) and willingness to participate in a colorectal cancer screening campaign. The mentioned factors are modifiable and therefore susceptible of intervention. In fact, interventional studies focused on average-risk population have tried to increase colorectal cancer screening uptake by improving public knowledge and modifying attitudes. In the present paper, we reviewed the factors impacting adherence to colorectal cancer screening and interventions targeting participants for increasing screening uptake.

## 1. Introduction

Colorectal cancer (CRC) is the fourth leading cause of death worldwide causing approximately 608.000 deaths in 2008 and 8% of all cancer related deaths. CRC also ranks as the third more incidental cancer accounting for 1.2 million new cases in 2008 [1].

In this way, professional organizations have published screening recommendations to guide clinical practice in average-risk population as well as in relatives (FDR) of patients with CRC [2–4]. Currently, annual or biannual fecal occult blood test (FOBT), colonoscopy every 10 years, and rectosigmoidoscopy every 5 years are the most frequent strategies. Evidence clearly suggests that CRC screening with any of the three recommended tests reduces CRC mortality [5–8]. However, high rates of participation are mandatory for the success of any screening campaign in terms of mortality reduction and cost effectiveness, particularly for those screening strategies requiring shorter screening intervals to be effective (such as the FOBT) [9].

Unfortunately, despite the efficacy of CRC screening in reducing incidence and mortality rates, screening uptake remains behind that of other screening-amenable cancers and rates continue to be low worldwide [10, 11]. Although, the American Cancer Society suggested a 75% screening uptake as an acceptable goal, current screening rates according to the recent published data of the National Health Interview Survey barely caught up the 58% [12, 13]. Conversely, a recent report of the European Commission proposed 45% as the minimum participation rate, although 65% has been recommended [14]. However, current participation rates are far from that goal ranging from 20% to 52% in those countries with an organized screening programme [15].

A bunch of factors of screening compliance have been reported over the last two decades of research [16]. Those factors can be grouped in patient level factors, including sociodemographic and psychosocial factors and healthcare system and provider factors [16].

In the same way, interventions for increasing CRC screening uptake have focused on different levels including patients, subjects in the workplace and community-based settings, and healthcare systems and providers. The present review is focused on patient factors and interventions targeting patients for increasing CRC screening uptake.

## 2. Factors Impacting Patient Adherence

The research on the factors that influence health behaviours (i.e., screening uptake) is of great importance when we are dealing with low rates of screening participation. A broad understanding of those factors is crucial to explain health behaviours and may provide a foundation for well-informed public health programs. It may also contribute to the design of more efficient intervention strategies in order to change screening behaviours [71]. Much research has been performed on factors that predict the likelihood of CRC screening [71, 72]. Those factors can be classified as nonmodifiable (i.e., patient characteristics) and modifiable. Although most studies have been focused on patient characteristics, modifiable factors may be much more interesting as they are susceptible to be changed. Theories of health behaviour or theoretical models have been developed to understand why people do or do not practice different health behaviours, identifying modifiable factors which may be plausible targets of interventional strategies [73, 74]. Therefore, theoretical models have a dual purpose, “explanatory” and “interventionist.” Nonmodifiable factors include demographics (age, sex, race, ethnicity, and marital status), income, educational level, medical insurance, family history, healthy behaviours (i.e., screening for other cancers) or risky behaviours (i.e., toxic habits, sedentary life), and access to care (i.e., proximity to healthcare facilities, a regular source of care) [9, 75]. Conversely, modifiable ones include patient knowledge about CRC and screening, attitudes (i.e., perceived benefits of screening and barriers against screening), and perception of risk for developing a CRC [76, 77].

The studies investigating predictive factors usually assessed the outcome of CRC screening using the recommended intervals proposed by the different medical societies for the screening in the average-risk population: FOBT in the last 1 or 2 years, colonoscopy within the past 10 years, or rectosigmoidoscopy within the past 5 years.

Recently, the 2010 NHIS survey on the use of CRC screening in the recommended intervals and individual related factors of screening participation in USA has been published [12]. The factors tested in this survey included mostly nonmodifiable factors. There were statistically significant differences (*P* < 0.05) in use in terms of age, race, education, income, type of healthcare insurance, usual source of healthcare, and family history of CRC. Others like physician recommendation [78, 79] and utilization of other preventive health services [80, 81] have been reported in several studies.

Screening rates gradually increased from 50 to 70 years. This relationship is in keeping with previous analysis reported by the NHIS in 2000 and 2005 [82, 83]. This finding could be explained because Medicare covers CRC screening in people older than 65 years. However, mixed results were found in recent European studies and therefore other factors may interfere with this association [18, 84]. For instance, whereas in a recent Spanish study carried out in nonselected participants older than 50 years [84], past screening uptake was higher among people younger than 65 years, in a randomized study performed in Italy [18], participants aged 65 years or older experienced higher rates of screening. Disparities have been found in some racial minorities in the USA. Although NHIS reports published in the last decade (including the last one) did not find any difference between whites and blacks, other minorities such as Asians and American Indians were less likely to report being screened than whites [12, 83]. In the same way, lower screening rates are consistently reported for Hispanics [85]. Cultural factors, low income, low-educational level, and the lack of healthcare insurance are known barriers against screening, being more prevalent in minority groups [85, 86].

Most studies agree that CRC screening is increased in highly educated participants. Literacy has been used as a variable to explain the impact of education on health behaviour in general and screening participation in particular [87, 88]. These findings are similar in European and in US studies [43, 85, 89]. As the educational level is usually lower in racial/ethnic minorities, low-income groups, and those without health insurance, these variables have been advocated as potential modifiers [90, 91]. One of the most important factors for predicting participation is socioeconomic deprivation, highlighted by the recent NHIS survey showing a progressive participation with a higher annual family income [12]. The importance of this factor is closely related to insurance status. In the NHIS survey, people with any type of insurance (private, military without private, and only government/public) were more likely to report being screened than those without. This factor is more important in countries in which health services are not government founded. Access of care defined as usual or regular source of care has been associated with higher rates of screening uptake. The pivotal role of primary care physician (PCP) for recommending and increasing CRC screening is well established [92, 93]. In a nationwide US survey conducted by the National Center for Health Statistics [12], 62% of those with usual source of care reported use of any test within the recommended intervals compared with only 22.4% without. This factor is in accordance with results in European studies [43]. Uninsured people might not have a regular healthcare provider and subsequently, they may not receive a referral for testing.

Several studies suggested that individuals with a family history of CRC are more likely to engage in screening than the average-risk population [9, 94]. In two recent studies carried out in Spain including 953 consecutive average-risk individuals and 334 relatives of patients with CRC [43, 95], 13% and 22%, respectively, had undergone a CRC screening test and only in 1% and 8%, respectively, the indication was screening. A subjective perception of higher risk of developing CRC was one of the most important predictors in the multivariate analysis [95]. Controversial results were obtained regarding gender across the studies. Although, in general, men have been more willing to participate than women [82, 96], recent data suggest that gender gap in CRC screening may be decreasing [12]. Marital status was consistently associated with CRC screening, either in USA or in Europe [82, 83, 97]. A large European study [97], controlled by age and educational level, showed a higher participation in married people and observed that invitations of both partners increased participation rates.

Health behaviours not related to CRC screening have been associated with CRC screening uptake [82, 83, 98]. A recent large multicenter study conducted in 15.000 adults aged 50–74 years in USA [98] assessed the risk factors of nonparticipation. In that study, nonwhite participants, with a low educational level, current smokers, and those who had lower rates of cancer screening (such as use of mammography, pap screening tests, or prostate-specific antigen test) were more likely to be nonparticipants.

The lack of knowledge about CRC and screening was reported as a prominent barrier for screening adherence [30, 82, 99]. Some authors suggested that it is more important in areas where screening is opportunistic [100]. Furthermore, knowledge about CRC has proven to be an independent predictor of positive attitudes toward screening, and both knowledge and attitudes are correlated with intention to be screened [101, 102]. Lack of knowledge is linked to low levels of education, minority ethnic groups, lack of health insurance, and low household income [76, 103, 104]. Different items have been used for assessing the level of knowledge, including signs and symptoms of CRC, risk factors, incidence, prognosis, and awareness of screening methods. In different studies carried out by the NHIS, the lack of knowledge of either FOBT or endoscopy as a screening test was a barrier, being reported by half of the participants [12, 82].

In a prospective study conducted in 953 average-risk participants [43], knowledge of CRC signs or symptoms was the strongest predictor for either having ever used a screening test (OR 6.46, CI 95% [4.28–9.74]) or being up-to-date with CRC screening (OR 7.23, CI 95% [4.36–11.98]).

Among the theoretical models of the health behaviour process, the Health Belief Model (HBM) remains one of the most popular models [72]. It has been used to examine cancer screening and other preventive behaviours as well as associations between health beliefs and the willingness to seek cancer screening services [105, 106]. The HBM measures people's beliefs regarding their risk for a health problem. According to this perception, HBM estimates the probability to take action to prevent, control, or screen for a disease and identifies specific constructs that may influence this behaviour [71]. The key constructs of perceived susceptibility and severity, perceived benefits and barriers, cues to action, and the more recent addition of self-efficacy, are the core constructs of the HBM. All these constructs are extremely interesting, as they are plausible targets of intervention.

High-risk perception has been cited frequently as a predictor of CRC screening. For instance, in a large representative sample of UK [107], participants who answered that their risk was higher than the average-risk population were more willing to participate in CRC screening (98%) than those who answered with the same risk (84%). In a Spanish study carried out in family-risk population [95], a high subjective perception of risk was an independent predictor for CRC screening (odds ratio = 2.87, 95% confidence interval: 1.10–7.46; *P* = 0.03). In another study [108], a higher perception of risk among relatives of patients with CRC increased with the number of relatives with CRC.

Negative attitudes or barriers such as anxiety, embarrassment, disinterest, fear of cancer or screening tests, lack of time, feeling healthy, subjective perception of pain or danger, discomfort, apprehensions about bowel preparation, and laxatives or insertion of the scope have been described across the studies [76, 109–111].

Conversely, potential participants with positive attitudes or perceived benefits of screening are more willing to participate in screening behaviours. Some of them include early cancer detection, regular checkups, or screening help to calm someone down [110].

A recent study conducted in Spain in average-risk population [110] showed that negative attitudes were more important than benefits in the health behaviour process. In that study, fear of CRC or screening tests and embarrassment were the main barriers that contributed to a low participation rate.

A systematic review about barriers and facilitators of CRC screening showed that the most commonly reported barriers related to screening tests were unpleasantness, discomfort, or perceived risk associated with performing the tests [112].

## 3. Interventions

We carried out a review searching in the PubMed and Cochrane library. The following MESH terms were used: “intervention,” “improvement,” “promotion,” “increasing,” and “colorectal cancer screening.” Inclusion criteria included (1) English language, (2) full manuscript publication, (3) CRC screening behaviors defined as completion of any CRC screening test, (4) randomized controlled trials comparing any type of intervention with the usual care or with other interventions; systematic reviews and the articles included as well as articles from references were retrieved; an additional search of manuscripts after the date of publication of the systematic reviews was performed, and (5) published before August, 2012.

The types of interventions focused on participants to increase CRC screening were selected according to the Guide to Community Preventive Services, and they included the following [113]:

- participant reminders: they included any material used to remind participants that they are due for CRC screening. Generally, reminders consisted of printed material (generally a mailed letter or postcard) or telephone messages;
- small media: informational or educational material delivered in pamphlets, brochures, leaflets, newsletters, letters, calendars, flip charts, or video about screening;
- one-to-one education: delivering information or providing motivation in an individual setting by health professionals or trained people (i.e., peer coaches and patient navigators);
- group education: delivering information about benefits, indications, and how to overcome barriers to screening and providing motivation in a group setting (usually lowincome communities or racial/ethnic minorities) by a health professional or trained people;
- reduction of structural barriers or reducing out-of-pocket costs: focused on decreasing economic and noneconomic obstacles to screening. They try to overcome access burdens (i.e., providing free transportation to patients, maildelivered FOBT, or free assistance by trained people, patient navigators);
- incentives: rewards that motivate people to accept or seek cancer screening. Although this category is also included, no RCT was found.

In most of the studies, several interventions were actually a combination of two or more interventions, specially, one-to-one or group interventions with small media or reminders or small media with reminders. So, it was difficult to assess the effect of each individual intervention in CRC screening behaviour. Studies from each category are compiled in Tables 1 to 5.

### 3.1. Patient Reminders

Twelve RCT were qualified for inclusion (Table 1) [17–28]. Most studies used more than one intervention for increasing participation rates. In brief, 10 used a combination of small media material and reminders [17–25, 27]. In 2 of them strategies to overcome structural barriers were also included [22, 26], and in 1 study one-to-one education strategy was included [26]. Only 1 study used just reminders [28], whereas in the study by Coronado et al. and Sequist et al. up to 2 different strategies along with reminders were used (strategies to overcome structural barriers plus one-to-one education and strategies to overcome structural barriers plus small media, resp.) [22, 26]. In 4 studies the outcome was just adherence to FOBT [20, 21, 23, 26], in 5 adherence with any CRC screening tests [17, 22, 24, 25, 27], in 2 colonoscopy completion rate [19, 28], and in 1 either flexible sigmoidoscopy or FOBT screening [18]. Overall the intervention using reminders increased participation in 8 studies (67%) ranging the effect compared with the usual care group from 5.7% to 35.7% [17, 19, 20, 22, 23, 25, 26, 28].

### 3.2. Small Media

Thirty RCT using small media based interventions for increasing participation rate were retrieved (Table 2) [17–25, 27, 29–32, 34–42, 44–48, 110, 114]. The studies by Ruffin et al. [41], Dolan and Frisina [32], Marcus et al. [38], Segnan et al. [18], Doorembos et al. [27], and Lipkus et al. [37] compared different small media strategies without the inclusion of a usual care group. In 3 of the 6 studies, a trend was found with the increased number of interventions [37, 38, 41], but no significant difference was found in the rest. In the remaining studies, small media interventions were compared with usual care. In 10 of these studies, several small media strategies were combined including educational leaflets, brochures, calendars, booklets, or videotapes [20, 21, 24, 25, 30, 31, 34, 35, 44, 47]. However, only in 4 of them (40%), at least one intervention group had a positive effect on participation [20, 25, 30, 35]. In the studies in which only one media intervention was used, 2 (29%) increased participation rate [33, 110]. Small media were combined with another strategy in 10 studies, in 5 of them only with reminders [19, 21, 24, 25, 48], in one with overcoming structural barriers [29], and in another one with one-to-one education [35]. In 3 studies, small media, overcoming structural barriers, and reminders were used altogether [17, 22, 23]. In 6 (60%) of the RCT, with a combination of small media with another strategy, the intervention had a positive effect on screening participation [17, 19, 22, 23, 25, 35].

Overall, in 12 studies (50%), participation rate was higher in the intervention group than in the usual care group [17, 19, 20, 22, 23, 25, 30, 33, 35, 41, 46, 110].

### 3.3. One-to-One Education

Fifteen RCT assessed the benefits of one-to-one strategy on screening participation rate (Table 3) [26, 42, 45, 46, 49–58, 115]. Trained health educators, patient navigators, community health workers, or health professionals (i.e., nurses or GPs) participated across the studies. In most of the studies, one-to-one strategy was combined with another one (in 5 studies with small media [49, 51, 52, 58, 115], in 2 studies with overcoming barrier strategies [55, 57], in three studies with both [42, 46, 54], and in 2 studies with overcoming barriers and reminders) [26, 53]. In 9 of the studies, the intervention was compared with usual care [42, 45, 46, 50, 52, 55, 56, 58]. In 6 of them (67%), the intervention increased participation rates compared with usual care [42, 46, 50, 52, 55, 56].

Overall, in 12 of the RCT (80%), the intervention increased participation rates [26, 42, 46, 50–57, 115].

### 3.4. Group Education

Six RCT used group education strategies, usually focused on low-income communities or ethnic/racial minorities (Table 4) [35, 57, 59–62]. In all the studies, another strategy was included, usually a small media intervention. Only in 3 studies, it was compared with usual care [35, 59, 61]. All of them showed a significant benefit of the intervention.

Overall, 5 studies (83%) showed a positive effect of the group educational intervention [35, 57, 59, 61, 62].

### 3.5. Reduction of Structural Barriers and Out-of-Pocket Costs

Nineteen studies assessed the usefulness of the reduction of structural barriers/out-of-pocket costs on CRC screening participation (Table 5) [17, 18, 22, 26, 42, 46, 50, 53–55, 57, 63–70]. Free posted FOBTs, prepaid postage, and assistance provided by health or nonhealth trained people (i.e., patient navigator, prevention care managers, or community health workers) were the most frequent strategies used. Fourteen studies combined different strategies, mainly with small media and/or reminders [17, 18, 22, 26, 42, 46, 50, 53–55, 57, 63, 66].

Ten of them compared this intervention with usual care [22, 26, 42, 46, 50, 55, 64, 68–70]. All of them showed a positive effect of the intervention (Table 1). Overall, only one study did not find significant differences in favour of the intervention [18].

## 4. Conclusion

High participation rates are of utmost importance for the success of any screening campaign. Efforts are needed to figure out modifiable and nonmodifiable factors impacting adherence in each specific population. Knowledge of modifiable factors is gaining special interest in order to design specific interventions. Educational interventions (one-to-one or group interventions), reminders encouraging screening, and interventions based on reduction of structural barriers and out-of-pocket costs seem to be the most effective patient level interventions for increasing participation rates.

## Figures and Tables

**Table 1:** Randomized controlled trials using patient reminders as interventional strategy.

Author (yr)	Aim	Intervention	Participants (*n*)	Participation, *n* (%)	Follow-up (months)
Church et al. (2004) [17]	Any screening test after intervention	Group 1: mailed FOBT and educational brochure	1255	13.2*	12
		Group 2: same as Group 1 and phone call reminder		14.1	
		Group 3: just mailed FOBT		7.8	

Segnan et al. (2005) [18]	Screening FS or FOBT after intervention	Group 1: mailed invitation to biennial FOBT, FOBT, instructions, educational leaflet, 2 reminder letters	26682	30	24
		Group 2: biennial FOBT delivered by the PCP or screening facility		28	
		Group 3: same as Group 1 and invitation to choose between FOBT and once only FS		27	
		Group 4: same as Group 1 and mailed invitation to FS		28	
		Group 5: same as Group 1 and invitation to FS followed by biennial FOBT		28	

Denberg et al. (2006) [19]	Screening colonoscopy after intervention	Group 1: mailed educational brochure with reminder after primary care visit	781	70.7*	4
		Group 2: usual care		59	

Myers et al. (2007) [20]	FOBT screening after intervention	Group 1: mailed letter, information booklet, FOBT, reminder letter	1546	46*	24
		Group 2: same as Group 1 and tailored message pages		44	
		Group 3: same as Group 2 and reminder phone call		48	
		Group 4: usual care		33	

Chan and Vernon (2008) [21]	FOBT screening after intervention	Group 1 (home e-mail access): e-mailed reminders, FOBT, last screening results, and link for additional information	97	26	18
		Group 2 (home e-mail access): usual care		23	
		Group 3 (public library access): same as Group 1		0	
		Group 4 (public library access): same as Group 2		33	

Sequist et al. (2009) [22]	Any screening test after intervention	Group 1: educational pamphlet, mailed FOBT with a stamped return envelope, telephone number to schedule colonoscopy or flexible sigmoidoscopy, and mailed reminder	21860	44*	15
		Group 2: usual care		38.1	

Lee et al. (2009) [23]	FOBT screening after intervention	Group 1: mailed educational reminder and FOBT	775	64.4*	6
		Group 2: usual care		48.4	

Sequist et al. (2011) [24]	Any screening test after intervention	Group 1: electronic reminders, educational information, and link for additional information	1103	15.8	4
		Group 2: usual care		13.1	

Cameron et al. (2011) [25]	Any screening test after intervention	Group 1: reminder letter, educational brochure, educational digital video disc, and phone call reminder	628	18.2*	6
		Group 2: usual care		12.1	

Coronado et al. (2011) [26]	Screening FOBT after intervention	Group 1: usual care	501	2*	9
		Group 2: mailed FOBT and instructions		26	
		Group 3: same as Group 2, phone call reminder and home visit by trained community members addressing strategies for CRC screening and definition of CRC		31	

Doorenbos et al. (2011) [27]	Any screening test after intervention	Group 1: reminders and e-mailed calendar with messages of CRC screening	5633	4.1	15
		Group 2: calendar without any messages		4.4	

Leffler et al. (2011) [28]	Screening colonoscopy after intervention	Group 1: automated reminder system (3 reminders)	830	33.5*	6
		Group 2: usual care		17.8	

*Intervention groups increased CRC screening rates compared with the usual care.

**Table 2:** Randomized controlled trials using small media as interventional strategy.

Author	Aim	Intervention	Participants, *n*	Participation, *n* (%)	Follow-up (months)
Hart et al. (1997) [29]	FOBT completion after intervention	Group 1: invitation letter with mailed FOBT, brochure with educational information about CRC and screening, and prepaid envelope to return FOBT	786	36 (men aged 61–65)	NR
				39 (men aged 66–70)	
				38 (women aged 61–65)	
				31 (women aged 66–70)	
		Group 2: invitation letter with mailed FOBT and prepaid envelope to return FOBT	785	27 (men aged 61–65)	
				23 (men aged 66–70)	
				36 (women aged 61–65)	
				31 (women aged 66–70)	

Pignone et al. (2000) [30]	CRC screening after intervention	Group 1: 11-minute video about colon cancer screening and color-coded educational brochure	125	37*	7
		Group 2: generic brochure on automobile safety	124	23	

Friedman et al. (2001) [31]	FOBT completion after intervention	Group 1: educational video about main topics about CRC, screening benefits and FOBT completion, educational brochure, and note card to order FOBT	110	44	2
		Group 2: same information given to Group 1 plus questionnaires before and after the video	50	36	
		Group 3: brochure plus note card to order screening			

Dolan and Frisina (2002) [32]	Any screening test after intervention	Group 1: decision aid about the different CRC screening tests available (Analytic Hierarchy Process)	45	40	6
		Group 2: one-to-one simple educational intervention, consisting of the explanation of the screening options	43	33	

Wardle et al. (2003) [33]	Flexible sigmoidoscopy screening after intervention	Group 1: mailed intervention brochure addressing barriers and benefits of flexible sigmoidoscopy screening	1453	54* (<0.05)	24
		Group 2: standard invitation group	1513	50	

Ruffin and Gorenflo (2004) [34]	Any screening test after intervention	Group 1: usual care	4212	FOBT 40.9; other 11	36
		Group 2: office intervention. To provide, at every patient encounter, past screening history and current screening recommendations for access by all the staff	4160	FOBT 24; other 13	
		Group 3: patient intervention. To provide patients the current screening guidelines and a record of their past screening and cues to the future	4286	FOBT 33.9; other 16	
		Group 4: Groups 2 and 3 combined	4557	FOBT 34 other 8	

Church et al. (2004) [17]	Any screening test after intervention	Group 1: mailed FOBT and educational brochure	1255	13.2*	12
		Group 2: same as Group 1 and phone call reminder		14.1	
		Group 3: just mailed FOBT		7.8	

Campbell et al. (2004) [35]	FOBT screening test after intervention	Group 1: tailored print and video intervention (educational newsletters, and targeted videotapes about beliefs, barriers, knowledge and motivation)	587	37*	12
		Group 2: Lay Health Advisor intervention (enhanced support for healthy eating, physical activity, and CRC screening)		33	
		Group 3: combined Group 1 and Group 2		31	
		Group 4: usual care		22	

Zapka et al. (2004) [36]	Any CRC screening test after intervention	Group 1: mailed CRC educational videotape	938	55^†^	6
		Group 2: usual care		55	

Lipkus et al. (2005) [37]	FOBT screening after intervention	Group 1: nontailored information including a brochure about CRC risk factors	216	60 (year 1); 52 (year 2) 41 (year 3)	36
		Group 2: nontailored information: same information given to Group 1 plus lifestyle information and occupational risk factors	212	60 (year 1); 54 (year 2) 59 (year 3)*	
		Group 3: Tailored information: same information given to Group 1 plus tailored information highlighting the risk factor which increases their personal CRC risk	218	68 (year 1); 57 (year 2) 49 (year 3)	
		Group 4: Tailored information: same information given to Group 3 plus counselling about lifestyles and occupational factors on CRC risk	214	74 (year 1)*; 59 (year 2) 51 (year 3)	

Segnan et al. (2005) [18]	Screening FS or FOBT after intervention	Group 1: mailed invitation to biennial FOBT, FOBT, instructions, educational leaflet, and 2 reminder letters	26682	30	24
		Group 2: biennial FOBT delivered by the PCP or screening facility		28	
		Group 3: same as Group 1 and invitation to choose between FOBT and once only FS		27	
		Group 4: same as Group 1 and mailed invitation to FS		28	
		Group 5: same as Group 1 and invitation to FS followed by biennial FOBT		28	

Marcus et al. (2005) [38]	Any CRC screening test	Group 1: educational message encouraging screening and single untailored mailed booklet	380	42	14
		Group 2: educational message encouraging screening and single tailored mailed booklet	377	44	
		Group 3: educational message encouraging screening and multiple tailored print materials using information at baseline mailed at different intervals	424	51**	
		Group 4: educational message encouraging screening and multiple tailored print materials using information at baseline and at 6 months. They were mailed at different intervals	419	48	

Miller et al. (2005) [39]	FOBT screening after intervention	Group 1: computer educational program about FOBT	93	62	1
		Group 2: participants received instructions by a nurse on how to complete FOBT	101	63	

Denberg et al. (2006) [19]	Screening colonoscopy after intervention	Group 1: mailed educational brochure with reminder after primary care visit	781	70.7*	4
		Group 2: usual care		59	

Myers et al. (2007) [20]	FOBT screening after intervention	Group 1: mailed letter, information booklet, FOBT, and reminder letter	1546	46*	24
		Group 2: same as Group 1 and tailored message pages		44	
		Group 3: same as Group 2 and reminder phone call		48	
		Group 4: usual care		33	

Costanza et al. (2007) [40]	Any CRC screening after intervention	Group 1: educational brochure and tailored computer assisted telephone counselling by trained interviewers	2448	25	22
		Group 2: usual care		24	

Ruffin et al. (2007) [41]	CRC screening after intervention	Group 1: interactive educational website about CRC tests	174	56*	6
		Group 2: same information given to group 1 but noninteractive		33	

Chan and Vernon (2008) [21]	FOBT screening after intervention	Group 1 (home e-mail access): e-mailed reminders, FOBT, last screening results, and link for additional information	97	26	18
		Group 2 (home e-mail access): usual care		23	
		Group 3 (public library access): same as Group 1		0	
		Group 4 (public library access): same as Group 2		33	

Sequist et al. (2009) [22]	Any screening test after intervention	Group 1: educational pamphlet, mailed FOBT with an stamped return envelope, telephone number to schedule colonoscopy or flexible sigmoidoscopy, and mailed reminder	21860	44*	15
		Group 2: usual care		38.1	

Lee et al. (2009) [23]	FOBT screening after intervention	Group 1: mailed educational reminder, mailed FOBT, and postage paid return envelope	775	64.4*	6
		Group 2: mailed FOBT and postage paid return envelope		48.4	

Percac-Lima et al. (2009) [42]	Colonoscopy screening after intervention	Group 1: introductory letter and language-concordant phone call	1223	27	9
		Group 2: usual care		12	

Gimeno-Garcia et al. (2009) [43]	FOBT after intervention	Group 1: CRC educational video	158	69.6*	2 weeks
		Group 2: nonmedical video		54.4	

Aragones et al. (2010) [44]	CRC screening after intervention	Group 1: CRC educational video and brochure	65	55	3
		Group 2: usual care		11	

Simon et al. (2010) [45]	CRC screening after intervention	Group 1: automated telephone with information for CRC screening	20938	30.6	12
		Group 2: usual care		30.4	

Lasser et al. (2011) [46]	Colonoscopy or FOBT screening after intervention	Group 1: introductory letter with educational material and telephone calls (language concordant)	465	33.6^∗‡^	12
		Group 2: usual care		20	

Sequist et al. (2011) [24]	Any screening test after intervention	Group 1: electronic reminders, educational information, and link for additional information	1103	15.8	4
		Group 2: usual care		13.1	

Vernon et al. (2011) [47]	CRC screening after intervention	Group 1: tailored group (narrative videos, list of common concerns about CRC screening, and patient-physician discussion)	413	28	6
		Group 2: website program (educational program developed by the Centers for Disease Control and Prevention)	398	31	
		Group 3: usual care	413	30	

Cameron et al. (2011) [25]	Any screening test after intervention	Group 1: reminder letter, educational brochure, educational digital video disc, and phone call reminder	628	18.2*	6
		Group 2: usual care		12.1	

Doorenbos et al. (2011) [27]	Any screening test after intervention	Group 1: reminders and e-mailed calendar with messages of CRC screening	5633	4.1	15
		Group 2: calendar without any message		4.4	

Misra et al. (2011) [48]	Any screening test after intervention	Group 1: usual care	1224	33.4^#^	12
		Group 2: web-based program (educational program developed by the Centers for Disease Control and Prevention)		35.4	
		Group 3: tailored interactive computer based intervention (based on the transtheoretical model)		32	

*Intervention groups increased CRC screening rates compared with the usual care.

**Intervention 3 versus Intervention 1, *P* = 0.03.

^†^Significantly higher participation in the group of participants who viewed the video.

^‡^More beneficial for patients whose primary language was not English.

^#^Despite the fact that there were no significant differences in participation, the usual care strategy was the most cost-effective strategy.

NR: nonreported.

**Table 3:** Randomized controlled trials using one to one education as interventional strategy.

Author	Aim	Intervention	Participants (*n*)	Participation, *n* (%)	Follow-up (month)
Gray and Pennington (2000) [49]	Flexible sigmoidoscopy after intervention	Group 1: invitation to have screening and explanatory leaflet	165	27	NR^#^
		Group 2: same as Group 1 and discussion with the general practitioner	154	21	

Stokamar et al. (2005)	FOBT completion after intervention	Group 1: educational sessions by primary care nurses, 2-page handout on CRC screening, AND instructions about stool collection	396	65.9*	6
		Group 2: instructions about stool collection	392	51.3	

Jandorf et al. (2005) [50]	CRC screening after intervention	Group 1: patient navigator (written reminders, phone calls, scheduling assistance, and encouraging participation)	38	23.7*	6
		Group 2: usual care	40	5	

Basch et al. (2006) [51]	CRC screening after intervention	Group 1: tailored telephone educational intervention based on behavioral sciences and educational theories	226	27*	12
		Group 2: printed materials (cover letter and brochure published by the Centers for Disease Control and Prevention)	230	6.1	

Tu et al. (2006) [52]	FOBT completion after intervention	Group 1: health educator and educational materials (video and motivational and informational pamphlet)	105	70*	6
		Group 2: usual care	105	28	

Dietrich et al. (2006) [53]	Any CRC screening after intervention	Group 1: educational brochure and 4 support calls by prevention care managers (provided motivational support, sent reminders and FOBT, arranged transportation, etc)	696	63* 43 (FOBT)	18
		Group 2: single telephone call answering questions about screening and recommendations to order screening	694	50 31 (FOBT)	

Dietrich et al. (2007) [54]	Any CRC screening after intervention	Group 1: trained bilingual outreach specialist (telephone calls recommending screening) and mailed educational material	653	25*	13
		Group 2: prevention care managers (telephone calls recommending screening and discussing barriers against screening), material about overcoming barriers, and support scheduling screening appointments	663	32	

Beach et al. (2007) [55]	Any CRC screening after intervention	Group 1: prevention care managers (telephone reminders, emotional support, overcoming screening barriers, and scheduling screening appointments)	528	53*	18
		Group 2: usual care	542	38	

Turner et al. (2008) [56]	Colonoscopy screening after intervention	Group 1: peer coach (patients with special training in communication skills) and phone calls	66	68.6**	19
		Group 2: brochures by the American Cancer Society and Centers for Disease Control and Prevention	70	57.6	
		Group 3: no support needed (patients with high readiness)	49	81.6	
		Group 4: not contacted	49	61.2	
		Group 5: declined support	41	48.8	

Percac-Lima et al. (2009) [42]	Any CRC screening after intervention	Group 1: introductory letter + educational material + phone or in-person contact by language-concordant navigator (community health workers)	409	27* 21 (colonoscopy)*	9
		Group 2: usual care	814	12 10 (colonoscopy)	

Blumenthal et al. (2010) [57]	CRC screening after intervention	Group 1: introductory meetings and written information (CRC screening information and National Cancer Institute pamphlet)	62	17.7	6
		Group 2: same as Group 1 and 3 sessions (45 minutes) with a health educator (one to one)	63	22.2	
		Group 3: same as Group 3 but in groups of 4–14 participants (group education)	67	25.4	
		Group 4: same as Group 1 and financial support covering (covering screening, transportation, and other nonmedical expenses)	65	33.9^∗†^	

Simon et al. (2010) [45]	Any CRC screening after intervention	Group 1: interactive outreach phone call to engage participants in conversation about CRC screening options and barriers against screening	10432	30.6	12
		Group 2: usual care	10506	30.4	

Menon et al. (2011) [58]	Any CRC screening after intervention	Group 1: counselling calls 2 weeks, 1 month, and 6 months after intervention and telephone based motivational interview with discussion about beliefs and stages of readiness about CRC screening	178	18.5	6
		Group 2: counselling calls 2 weeks, 1 month, and 6 months, postintervention tailored health counselling (standardized survey over the phone, computer program with tailored messages, and phone call by a trained interventionist)	168	23.8	
		Group 3: usual care	169	11.8	

Lasser et al. (2011) [46]	Colonoscopy or FOBT screening after intervention	Group 1: introductory letter with educational material and patient navigation-based intervention (phone calls offering CRC screening)	235	33.6^∗‡^	12
		Group 2: usual care	230	20	

Coronado et al. (2011) [26]	Screening FOBT after intervention	Group 1: usual care	195	2*	9
		Group 2: mailed FOBT and instructions	168	26	
		Group 3: same as Group 2, phone call reminder and home visit by trained community members addressing strategies for CRC screening and definition of CRC	168	31	

^#^Nonreported.

*Statistically significant (*P* < 0.05).

**No comparisons between intervention groups and nonintervention groups. Nonsignificant differences between the 2 intervention groups. Significant difference in colonoscopy attendance in the no support needed group compared with those who declined support.

^†^Statistically significant Group 4 versus Group 1.

^‡^More beneficial for patients whose primary language was not English.

**Table 4:** Randomized controlled trials using group education as interventional strategy.

Author	Aim	Intervention	Participants, (*n*)	Participation, *n* (%)	Follow-up (month)
Powe et al. (2004) [59]	FOBT screening after intervention	Citizen centers			12
		Group 1: educational video, calendar, and poster brochure flier	134	61*	
		Group 2: educational video		46		
		Group 3: usual care		15		

Campbell et al. (2004) [35]	FOBT screening test after intervention	Group 1: tailored print and video intervention (educational newsletters and targeted videotapes about beliefs, barriers, knowledge, and motivation)	587	37*	12
		Group 2: Lay Health Advisor intervention (enhanced support for healthy eating, physical activity, and CRC screening)		33	
		Group 3: combined Group 1 and Group 2		31	
		Group 4: usual care		22	

Braun et al. (2005) [60]	FOBT screening after intervention	Group 1: CRC information given by native physician and a native CRC survivor who told his own experience	121	33	6
		Group 2: CRC information given by a nonnative nurse and brochure		40	

Blumenthal et al. (2010) [57]	CRC screening after intervention	Group 1: introductory meetings and written information (CRC screening information and National Cancer Institute pamphlet)	257	17.7	6
		Group 2: same as Group 1 and 3 sessions (45 minutes) with a health educator (one to one)		22.2	
		Group 3: same as Group 3 but in Groups of 4–14 participants (group education)		25.4	
		Group 4: same as Group 1, and financial support covering (covering screening, transportation and other nonmedical expenses)		33.9^∗†^	

Maxwell et al. (2010) [61]	FOBT screening after intervention	Group 1: small group educational session on CRC screening by a trained health professional, print take home material + free FOBT	547	30*	6
		Group 2: same as Group 1 but no free FOBT		25	
		Group 3: usual care		9	

Morgan et al. (2010) [62]	Colonoscopy uptake and knowledge after intervention	Group 1: long educational program on CRC (90-minute culturally targeted educational program including educational material, brochure, video, incentives, and phone calls reminders)	539	25.5*	3
		Group 2: abbreviated educational intervention		4.4	

*Statistically significant (*P* < 0.05).

^†^Statistically significant Group 4 versus Group 1.

**Table 5:** Randomized controlled trials for reduction of structural barriers and out-of-pocket costs as interventional strategy.

Author	Aim	Intervention	Participants (*n*)	Participation, *n* (%)
Mant et al. (1992) [63]	FOBT completion after intervention	Group 1: posted FOBT	404	25.5
		Group 2: posted FOBT and invitation to health check	397	31.7*
		Group 3: invitation to health check explaining that a FOBT would be offered at the appointment	402	20.6

Miller and Wong (1993) [64]	FOBT completion after intervention	Group 1: prepared FOBT kits with prepaid postage	159	74*
		Group 2: usual care	166	61

Freedman and Mitchell (1994) [65]	FOBT completion after intervention	Group 1: returning FOBT by hand	146	37
		Group 2: returning FOBT by mail		57
		Group 3: returning FOBT with prepaid postage		71*

Ore et al. (2001) [66]	FOBT completion after intervention	Group 1: mailed FOBT kit and information leaflet about CRC risks and importance of early detection	976	19.9*
		Group 2: FOBT kit request and information leaflet about CRC risks and importance of early detection	864	15.9

Courtier et al. (2002) [67]	FOBT completion after intervention	Group 1: mail invitation letter together with two containers for faecal sample collection	1060	68*
		Group 2: visit by a trained nonhealth professional who helped to collect the faecal sample	965	75

Church et al. (2004) [17]	Any screening test after intervention	Group 1: mailed FOBT and educational brochure	1255	13.2*
		Group 2: same as Group 1 and phone call reminder		14.1
		Group 3: just mailed FOBT		7.8

Segnan et al. (2005) [18]	Screening FS or FOBT after intervention	Group 1: mailed invitation to biennial FOBT, FOBT, instructions, educational leaflet, and 2 reminder letters	26682	30
		Group 2: biennial FOBT delivered by the PCP or screening facility		28
		Group 3: same as Group 1 and invitation to choose between FOBT and once only FS		27
		Group 4: same as Group 1 and mailed invitation to FS		28
		Group 5: same as Group 1 and invitation to FS followed by biennial FOBT		28

Jandorf et al. (2005) [50]	CRC screening after intervention	Group 1: patient navigator (written reminders, phone calls, scheduling assistance, and encouraging participation)	38	23.7*
		Group 2: usual care	40	5

Dietrich et al. (2006) [53]	Any CRC screening after intervention	Group 1: educational brochure and 4 support calls by prevention care managers (provided motivational support, sent reminders and FOBT, arranged transportation, etc.)	696	63* 43 (FOBT)
		Group 2: single telephone call answering questions about screening and recommendations to order screening	694	50 31 (FOBT)

Beach et al. (2007) [55]	Any CRC screening after intervention	Group 1: prevention care managers (telephone reminders, emotional support, overcoming screening barriers, and scheduling screening appointments)	528	53*
		Group 2: usual care	542	38

Dietrich et al. (2007) [54]	Any CRC screening after intervention	Group 1: trained bilingual outreach specialist (telephone calls recommending screening) and mailed educational material	653	25*
		Group 2: prevention care managers (telephone calls recommending screening and discussing barriers against screening), material about overcoming barriers, and support scheduling screening appointments	663	32

Percac-Lima et al. (2009) [42]	Any CRC screening after intervention	Group 1: introductory letter + educational material + phone or in-person contact by language-concordant navigator (community health workers)	409	27* 21 (colonoscopy)*
		Group 2: usual care	814	12 10 (colonoscopy)

Sequist et al. (2009) [22]	Any screening test after intervention	Group 1: educational pamphlet, mailed FOBT with a stamped return envelope, telephone number to schedule colonoscopy or flexible sigmoidoscopy, and mailed reminder	10930	44*
		Group 2: usual care	10930	38.1

Potter et al. (2009) [68]	Screening FOBT after intervention	Group 1: influenza vaccination, colourful multilingual education sheet and FOBT kit, and stamped return envelope.	246	57.3
		Group 2: influenza vaccination (usual care)	268	84.3

Blumenthal et al. (2010) [57]	CRC screening after intervention	Group 1: introductory meetings and written information (CRC screening information and National Cancer Institute pamphlet)	62	17.7
		Group 2: same as group 1 and 3 sessions (45 minutes) with a health educator (one-to-one)	63	22.2
		Group 3: same as Group 3 but in groups of 4–14 participants (group education)	67	25.4
		Group 4: same as Group 1 and financial support covering (covering screening, transportation, and other nonmedical expenses)	65	33.9^∗†^

Lasser et al. (2011) [46]	Colonoscopy or FOBT screening after intervention	Group 1: introductory letter with educational material and patient navigation-based intervention (phone calls offering CRC screening)	235	33.6^∗‡^
		Group 2: usual care	230	20

Coronado et al. (2011) [26]	Screening FOBT after intervention	Group 1: usual care	195	2*
		Group 2: mailed FOBT and instructions	168	26
		Group 3: same as Group 2, phone call reminder and home visit by trained community members addressing strategies for CRC screening and definition of CRC	168	31

Hoffman et al. (2011) [69]	FOBT/colonoscopy screening after intervention	Group 1: electronic medical record, and mailed gFOBT	3221	48.5*
		Groups 2, 3, and 4: CRC screening offered in a clinic	3184	18.6; 14.3; 18.8

Potter et al. (2011) [70]	CRC screening during Flu campaigns	Group 1: nurse offering FOBT to patients waiting for their influenza vaccination and an envelope for mailing the FOBT kits, and a reminder 1 month later	695	30.3*
		Group 2: usual care (only received influenza vaccination)	677	13.7

*Statistically significant (*P* < 0.05).

^†^Statistically significant Group 4 versus Group 1.

^‡^More beneficial for patients whose primary language was not English.
